# Effectiveness of Inferior Mesenteric Artery Embolization on Type II Endoleak-Related Complications after Endovascular Aortic Repair (EVAR): Systematic Review and Meta-Analysis

**DOI:** 10.3390/jcm11185491

**Published:** 2022-09-19

**Authors:** Natalia Niklas, Michalina Malec, Piotr Gutowski, Arkadiusz Kazimierczak, Paweł Rynio

**Affiliations:** Department of Vascular Surgery, Pomeranian Medical University in Szczecin, Al. Powstańców Wielkopolskich 72, 70-111 Szczecin, Poland

**Keywords:** inferior mesenteric artery, embolization, type II endoleak

## Abstract

Type II endoleak is one of the most common and problematic complications after endovascular aneurysm repair. It has been suggested that the inferior mesenteric artery (IMA) embolization could prevent further adverse events and postoperative complications. This article is a systematic review and meta-analysis following PRISMA guidelines. The Medline, PubMed, Embase, and Cochrane databases were used to identify studies that investigated the effect of IMA embolization on the occurrence of type II endoleaks and secondary interventions in a group of patients with abdominal aortic aneurysm who underwent EVAR compared with results after EVAR procedure without embolization. A random effects meta-analysis was performed. Of 3510 studies, 6 studies involving 659 patients were included. Meta-analysis of all studies showed that the rate of secondary interventions was smaller in patients with IMA embolization (OR, 0.17; SE, 0.45; 95% CI, 0.07 to 0.41; *p* < 0.01; *I*^2^ = 0%). The occurrence of type II endoleaks was also smaller in the embolization group (OR, 0.37; SE, 0.21; 95% CI, 0.25 to 0.57; *p* < 0.01; *I*^2^ = 16.20%). This meta-analysis suggests that IMA embolization correlates with lower rates of type II endoleaks and secondary interventions.

## 1. Introduction

In current clinical practice, endovascular aortic repair (EVAR) has become the most common method for abdominal aortic aneurysm treatment, and it is associated with fewer perioperative complications and a higher survival rate until three years postoperatively [1].

However, the most frequent complication following EVAR includes type II endoleaks, originating from collateral vessels, which consequently leads to a higher rate of secondary interventions [2]. It has been suggested that preoperative embolization of the inferior mesenteric artery (IMA) could prevent the development of type II endoleaks and reduce the frequency of secondary interventions [3]. Nevertheless, controversies exist, and, based on the results of a few studies, the benefits of prophylactic collateral vessel embolization before EVAR have not yet been fully proven and require further research [4,5]. The rate of technical success of the embolization procedure is variable. Usually, it relates to a longer duration of the operation, and there are issues arising around the cost-effectiveness of this procedure.

Nevertheless, it is suggested that routine preoperative IMA embolization in certain groups of patients could be an effective therapeutic strategy to prevent type II endoleak; however, further studies are needed [6]. Several meta-analyses and systematic reviews have already raised the issue of the effect of IMA embolization on complications after EVAR. As the last meta-analysis was conducted more than five years ago, there is a need to update the topic with the most up-to-date scientific knowledge [4]. More recent studies also analyzed the effect of sac embolization [7,8] or aortic side branches other than the IMA [9]. Meanwhile, in this research, we focused only on the embolization of the inferior mesenteric artery.

This meta-analysis aimed to investigate (in groups of patients with abdominal aortic aneurysm who underwent EVAR repair) the effect of inferior mesenteric artery embolization on type II endoleak-related complications compared with an EVAR procedure without IMA embolization.

## 2. Materials and Methods

### 2.1. Literature Search and Inclusion Criteria 

This systematic review was conducted according to Preferred Reporting Items for Systematic Reviews and Meta-Analyses (PRISMA) statement guidelines [10]. The study protocol for this research was not preregistered with any database. We searched the Ovid versions of MEDLINE, PubMed, Embase, and the Cochrane Library to identify studies investigating the association between IMA embolization and the occurrence of type II endoleaks and secondary reinterventions. The first database search was conducted on October 19th, 2020. The following combination of keywords was used connected through Boolean operators to maximize our search sensitivity: “embolization” AND” inferior mesenteric artery” OR “EVAR” AND “embolization” OR “endovascular aortic repair” AND embolization OR “EVAR” AND “coil” OR “endovascular aortic repair” AND “coil” The last database search and the abstract and topic analysis were finished on 24 August 2022, without any language limitations. Eligibility of the studies was checked by resorting to the PICOS (population, intervention, comparators/controls, outcomes, and study design) question [11], as follows:

- Population: Patients with abdominal aortic aneurysm who underwent EVAR.
- Intervention: Embolization of only inferior mesenteric artery without any additional embolization of aneurysm sac or other aortic side branches.
- Comparators/controls: Patients who underwent EVAR procedure without IMA embolization.
- Outcomes: The rate of type II endoleaks and secondary reinterventions.
- Study design: Observational case-control studies and randomized controlled trials were included.

Reviews, meta-analyses, and case reports were excluded from our research. Secondary reinterventions were defined as procedures used to treat type II endoleak-related complications. Studies needed to contain information about stent-graft implantation only for abdominal aortic aneurysm, excluding complex aortic repairs. The follow-up period was in the range of 6 months to 2.5 years. Titles and abstracts were studied to identify the articles compatible with our systematic review. The abstracts which passed the initial screening underwent further full-text review. The eligibility of the studies against our inclusion criteria was evaluated by two authors independently (N.N. and M.M.), and potential discrepancies were resolved by the third author (P.R.). 

### 2.2. Data Extraction and Assessment of Strengths and Weaknesses of Included Studies

Data from the included studies were extracted by three authors independently (N.N., M.M., and P.R.) in predefined tables. The following data were collected: sample sizes for the IMA embolization group and control group; study design; the presence of type II endoleak; secondary interventions related to type II endoleak. The risk of bias was assessed independently by two authors (N.N. and P.R.). A study assessment tool was created to determine the risk of bias in the included studies adapted from previously reported quality assessment tools and Standard Quality Assessment Criteria for Evaluating Primary Research Papers [12]. Additional criteria of the included studies relevant to this systematic review were also assessed. This included evaluating whether a technical aspect of IMA embolization was well-described (e.g., device used for the IMA embolization, description of the method, the rate of technical success). The definition of patent IMA and inclusion of CT scan before IMA embolization and during follow-up were also included. Importantly, we also assessed whether patients’ characteristics were present in each study and if the potential risk factors of embolization failure were reported (e.g., use of anticoagulants). Details regarding the quality assessment criteria can be found in Table 1.

### 2.3. Statistical Analysis

For all included papers, the embolization and non-embolization study group sizes were retrieved as well the type II endoleak presence during follow-up and the need for reintervention. These served for an odds ratio (OR) calculation for specific papers and as a summary of them all. The OR for the absence of type II endoleak and lack of reintervention were presented on forest plots. The *I*^2^ index was used to measure interstudy heterogeneity, and values below 25%, between 25% and 75%, and beyond 75% were regarded as indicating low, moderate, and high heterogeneity, respectively. A *p*-value < 0.05 was considered as statistically significant. A meta-analysis with random effects was performed. The publication bias was evaluated through visual inspection of the funnel plot for asymmetry. The assessment of the publication bias was conducted with Egger’s regression asymmetry. The analysis was performed in PQStat (PQStat Software 2022, v.1.8.4. Poznan, Poland)

## 3. Results

### 3.1. Study Identification

After removing of duplicates, the database yielded 1929 studies. The initial title and abstract screening were performed and 32 studies were included. Only 16 studies had full texts available, and they were evaluated against the inclusion criteria. The main reasons for the lack of availability of full-text PDFs were: (1) articles had a published abstract but were still in press and (2) studies had abstracts that were in English but the full-text PDFs were only available in languages other than English. Ten studies were excluded after full-text analysis (Table 2). 

**Figure 1:**
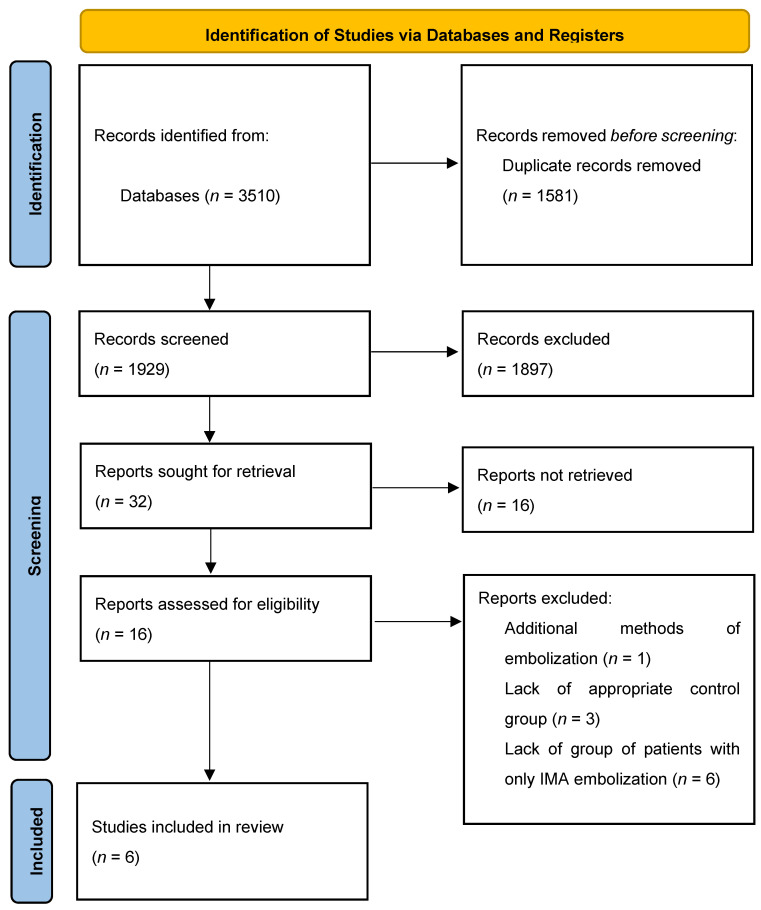
PRISMA 2020 flow diagram for new systematic reviews which included searches of databases only. PRISMA, Preferred Reporting Items for Systematic Reviews and Meta-Analyses.

Finally, six studied were included in the systematic review [22,23,24,25,26,27] (Figure 1). 

### 3.2. Study Characteristics

A total of 659 patients were included: 269 within embolization group and 390 in the control group (without IMA embolization). Five studies were retrospective, only one was a randomized controlled trial [22], and sample sizes ranged between 63 and 266. The included studies were published between 2001 and 2019. Two studies were performed in the United States [23,25] and the remaining studies were conducted in the United Kingdom [27], France [26], Finland [24], and Japan [22]. The inclusion criteria varied between studies. The major inclusion criterion for IMA embolization was visualization of patent IMA on preprocedural computed tomographic (CT) angiography [22,23,24,25,26,27]. In addition, conventional angiography was required in some studies [23,25,27]. Ward et al. [23] claimed that retrograde filling of IMA via collateral vessels and a stenotic origin that prevented visualization were responsible for the lack of visualization of the IMA on conventional angiography. Vaillant et al. [26] included patients with IMA > 3 mm without signs of occlusion or stenosis, whereas Samura et al. [22] prospectively randomized patients to receive EVAR with IMA embolization if they were at high risk of type II endoleak (IMA patency with IMA ≥ 3 mm, LAs ≥ 2 mm, or an aortoiliac-type aneurysm). Further details of the inclusion criteria can be found in Table 3. 

The principal method for IMA embolization was coils in five studies [23,24,25,26]. The coils used were interlocking detachable coils (Boston Scientific) [24], platinum microcoils (Boston Scientific, Natick, MA) [25], and regular coils (Cook, Letchworth, UK; Target, San Jose, CA) [27]. Vaillant et al. [26] in addition to coils used also plugs for IMA embolization. Samura et al. [22] performed the IMA embolization during EVAR using an Amplatzer Plug (AVP; St. Jude Medical, Plymouth, MN) in most patients and metallic coils in the remaining patients. The rate of technical success ranged between 68% and 100%. The amount of the iodinated contrast used for obtaining CT angiography varied from 100 ml to 150 ml between studies. The average operative time was mentioned only in two studies. For Samura et al. [22], the average operative time for the embolization group was 3.6 h and for the control group 2.9 h. Nevala et al. [24] reported that the operative time was 3.8 h vs. 4.3 h for the embolization and control group, respectively.

### 3.3. Patients’ Characteristics

Patients’ characteristics were variably reported. Basic characteristics are summarized in Table 4.

One study did not report patients’ characteristics [27]. Axelrod et al. [25] did not provide sufficient patient data in their work (they only provided the sex and age of patients); therefore, this study was not included in the table. The average age of participants ranged between 71 and 77 years [22,23,24,25,26]. The proportions of men in the embolization group and control group were 85 to 97% and 84 to 100%, respectively. Mean aneurysm diameter reported varied between 53 mm to 63 mm in the embolization group and 50 mm to 58 mm in the non-embolization group. Two studies reported no significant difference in age, sex, and maximal aneurysm diameter between groups [23,26]. Two studies included the number of patent lumbar arteries observed on preprocedural CT angiography, which was significantly higher in the IMA embolization group [23], while in the other study the results were not statistically significant [26]. The proportion of participants who were current or previous smokers ranged from 32% to 70%; however, three studies did not report smoking history [23,25,27]. There were not significant differences in the proportion of patients with diabetes, hypertension, coronary artery disease. and dyslipidemia between the embolization and no embolization groups [22,26]. However, in one study there was a statistically significant difference between the patients with hypertension, with the percentage higher in the IMA embolization group [24]. Only two studies reported the use of anticoagulants or antiplatelet agents in patients’ characteristics. Samura et al. [22] reported that 5.7% of patients in the embolization group and 9.4% in the non-embolization group used anticoagulants and 28.3% and 26.4% used the antiplatelet agents in the embolization vs. non-embolization groups, respectively. In the second study. 10.8% in the embolization group and 22.2% in the control group used anticoagulants [26]. The results were not statistically significant in either study. 

### 3.4. Risk of Bias Assessment

The strengths and weaknesses of the included studies are reported in Table 5. 

Five studies were of a retrospective design [23,24,25,26,27], and only one was a randomized controlled trial [22]. As a result, certain important patient factors which could contribute to the occurrence of type II endoleak were not available. The use of anticoagulants was not mentioned in four studies [23,24,25,27]. The criteria that allowed patients to undergo embolization with well-defined high-risk patients for the occurrence of type II endoleak (IMA patency with IMA ≥ 3 mm, Las ≥ 2 mm, or aortoiliac-type aneurysm) were included in one study [22]. In two studies, the decision about secondary intervention was made based on persistent type II endoleak and aneurysm sac enlargement greater than 5 mm [25,26]. In contrast, the presence of type II endoleak was a sufficient criterion for reintervention in three studies [23,24,27]. The reinterventions included endovascular embolizations as well as available conversions. Only percutaneous coil embolizations were noted in one study [25]. Vaillant et al. [26] reported multiple reinterventions in the control group, where 14 patients underwent 31 reinterventions. The stent-graft used for EVAR implantation and operative technique were well-described in all studies. Three-phase CT was performed to detect the present of endoleak at follow-up. In two studies there was a large disproportion in follow-up between the IMA embolization group and control group (21.41 months vs. 57.16 months and 985 days vs. 645 days, respectively) [23,26]. 

### 3.5. Data synthesis

**Figure 2:**
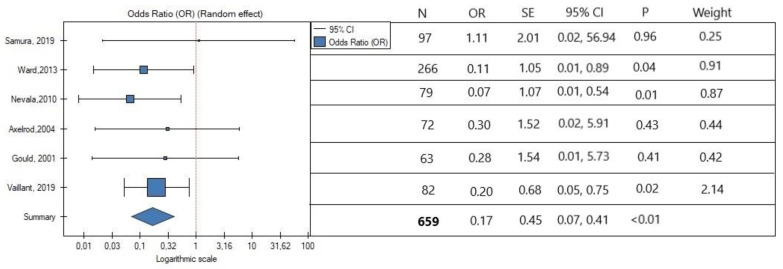
Association between IMA embolization and rate of secondary reinterventions. The horizontal line represents 95% confidence intervals (CIs). N, number of patients; OR, odds ratio; SE, standard error. Data referred from reference [22,23,24,25,26,27].

**Figure 3:**
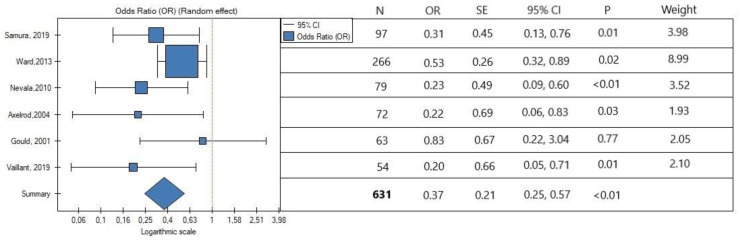
Association between IMA embolization and occurrence of endoleak type II. The horizontal line illustrates 95% confidence intervals (CIs). N, number of patients; OR, odds ratio; SE, standard error. Data referred from reference [22,23,24,25,26,27].

In the meta-analysis, the rate of secondary reintervention was lower in patients with intraoperative IMA embolization (OR, 0.17; SE, 0.45; 95% CI, 0.07 to 0.41; *p <* 0.01; Figure 2). Low heterogeneity was observed (*I*^2^ = 0%). The occurrence of type II endoleak was also lower in the IMA embolization group (OR, 0.37; SE, 0.21; 95% CI, 0.25 to 0.57; *p <* 0.01; Figure 3). Interstudy heterogeneity was also low (*I*^2^ = 16.20%). Overall, the embolization of IMA lowers the risk of type II endoleak occurrence by three times.

**Figure 4:**
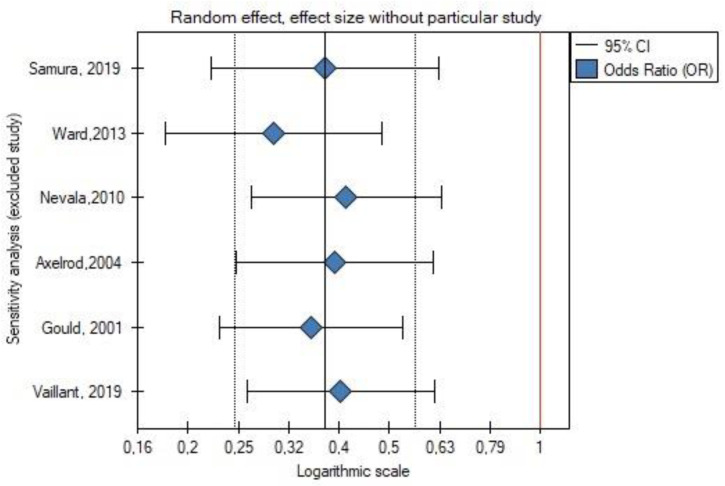
Leave-one-out meta-analysis for the occurrence of type II endoleak. Data referred from reference [22,23,24,25,26,27].

**Figure 5:**
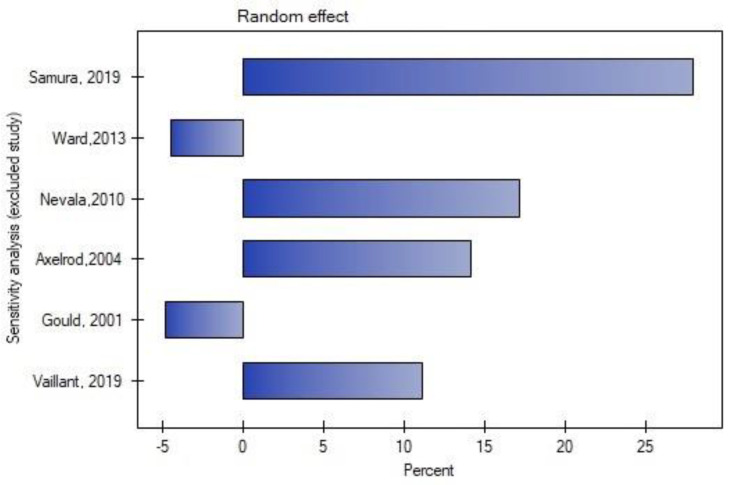
Leave-one-out meta-analysis with random effect and change of precision for the occurrence of type II endoleak. Data referred from reference [22,23,24,25,26,27].

**Figure 6:**
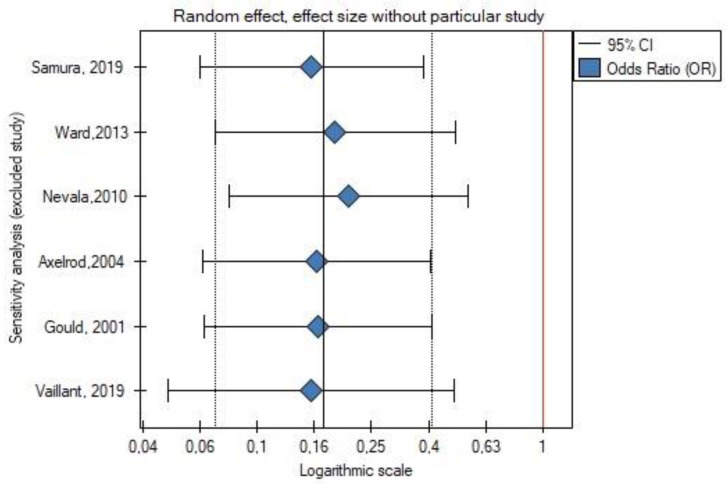
Leave-one-out meta-analysis for the rate of secondary reinterventions. Data referred from reference [22,23,24,25,26,27].

**Figure 7:**
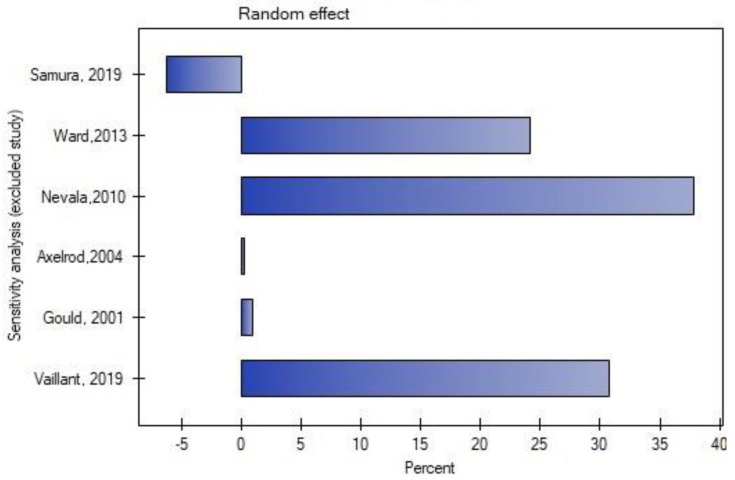
Leave-one-out meta-analysis with random effect and change of precision for the rate of secondary reinterventions. Data referred from reference [22,23,24,25,26,27].

Leave-one-out analysis was performed, and exclusion of certain studies did not significantly change the meta-analysis result for the occurrence of type II endoleak (Figure 4 and Figure 5) or for the rate of secondary reinterventions (Figure 6 and Figure 7).

### 3.6. Publication Bias

**Figure 8:**
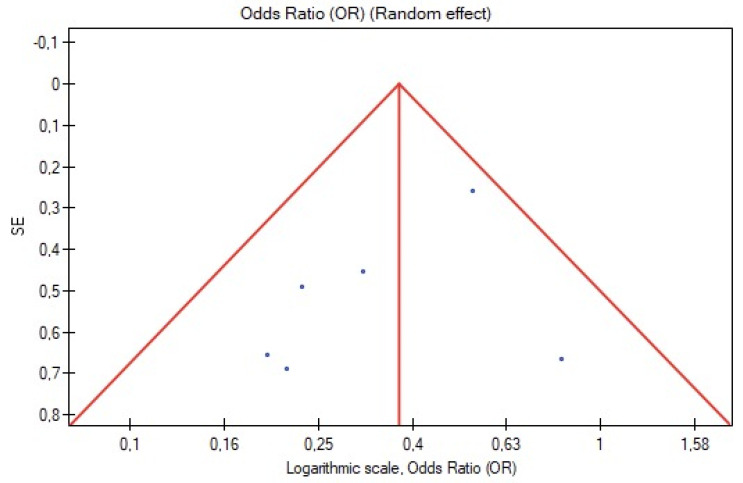
Funnel plot for the occurrence of type II endoleak.

**Figure 9:**
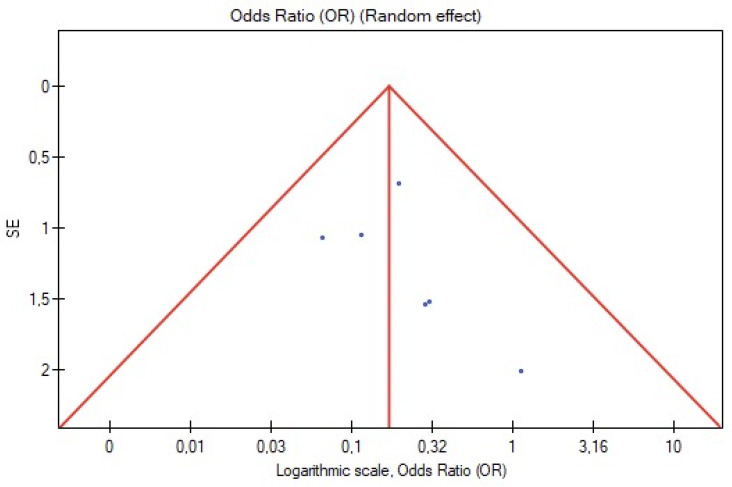
Funnel plot for secondary reinterventions.

Visual inspection of funnel plot symmetry insinuated no potential publication bias for the occurrence of type II endoleak and rate of reinterventions (Figure 8 and Figure 9). Furthermore, Egger’s linear regression test was not statistically significant for the occurrence of type II endoleak (Egger’s coefficient, −1.50; 95% CI, −4.57 to 1.57; *p* = 0.25) and secondary reinterventions (Egger’s coefficient, 0.76; 95% CI, −1.39 to 2.91; *p* = 0.38).

## 4. Discussion

This meta-analysis suggests that IMA embolization is associated with a lower rate of secondary reinterventions after EVAR and type II endoleaks. 

In recent years, EVAR has become the preferred method for abdominal aortic aneurysm (AAA) repair, as it is less invasive compared with open AAA repair and is associated with reduced early total and aneurysm-related mortality [28]. Nevertheless, patients treated with EVAR without IMA embolization have a higher rate of reinterventions and graft-related complications [29]. One of the most frequent complications which needs to be resolved is the occurrence of type II endoleak after EVAR [30], which results from retrograde perfusion into the aneurysmal sac from the side branches, such as the inferior mesenteric artery (IMA) and the lumbar arteries (LAs). The management of type II endoleaks remains controversial. In most patients, type II endoleak resolves spontaneously during the first 6 months after EVAR, and the treatment during this time is not necessary [4]. On the other hand, persistent type II endoleak, which lasts over six months, is correlated with an increased risk of adverse effects, including sac enlargement, reintervention, conversion to open repair, and even rupture [31]. In recent years, the idea of preventing type II endoleak rather than treating it has become more popular. The procedure of IMA embolization has also been shown to be effective in the prevention of type II endoleak in a different meta-analysis [8]. Moreover, in the most recent meta-analysis, authors concluded that prophylactic embolization during EVAR effectively prevents type II endoleak and reduces the reintervention rate. IMA embolization was mainly connected with a lower risk of secondary interventions, and non-selective embolization of aortic side branches showed a stronger relationship with a lower incidence of type II endoleak. Aneurysm sac coil embolization enhanced the clinical outcomes of EVAR; however, it showed no advantage over the IMA embolization [7]. There are few studies comparing the effectiveness of sac embolization with sole embolization of the inferior mesenteric artery and whether this combination could give a greater therapeutic effect. Further prospective randomized studies could help to establish more reliable conclusions.

There are many limitations of the IMA embolization method that need to be addressed. The principal problem is the proper qualification of patients to undergo IMA embolization. Even though risk factors for type II endoleak are well-established [32,33], in our meta-analysis, they were fully included in only one study [22]. Two studies reported the cost-effectiveness of the IMA embolization in selected group of patients [22,26]. Vaillant et al. [26] reported the cost of EUR 13.016 vs. EUR 12.063 for embolization vs. non-embolization groups, respectively, whereas Samura et al. [22] calculated all expenses in Japanese yen. After conversion to euros, the average cost of procedures in patients seems to be EUR 15 551.91 within the embolization group and EUR 14 572.09 for the control group. These two studies claimed that the procedure of IMA embolization during EVAR is a safe as well as cost-effective method. IMA embolization can be related to several serious complications in the postoperative period. Nevertheless, only one study reported a colonic infarction in a patient within 12 h of embolization that resulted in mortality [23]. Since IMA embolization is usually associated with increased use of the contrast agent [22], acute kidney injury can appear [26]. Other major complications after the IMA embolization procedure were not reported. 

There are multiple approaches to the management of type II endoleak after EVAR. The conservative treatment is initially preferred as some of the endoleaks tend to resolve spontaneously up to 12 months [34]. If necessary, most secondary interventions were transarterial embolization of aortic side branches. The biggest challenge reported was the occurrence of multiple reinterventions, which happened in a control group. Vaillant et al. [26] noted a secondary failure rate, defined as a reappearance of a type II endoleak, as high as 65% (13 out of 20 patients). Open conversions were also performed. Indeed, it is a tremendous clinical challenge since any additional reintervention puts patients at risk of postoperative complications; for example, exposure of patients to an increased amount of contrast can be associated with an increased risk of acute kidney injury [35]. 

Based on the available information, the most appropriate indication for IMA embolization seems to be: patent IMA visualized on preoperative computed tomography and subsequent conventional angiography and, favorably, IMA diameter > 3 mm. 

Although it has been suggested that IMA embolization during EVAR is associated with a lower rate of postoperative adverse effects, further studies are needed. Particularly, better identification of risk factors that put patients at high risk for occurrence of type II endoleak to properly select the group of patients for IMA embolization is required. The cost-effectiveness of the embolization procedure also needs to be clarified. More randomized controlled trials are needed to study the importance of IMA embolization. Due to the small number of RCT studies, we had to include more observational research in this meta-analysis. However, to make a well-designed study, the groups of patients should be matched in terms of demographic and clinical characteristics (for example, the use of anticoagulants). Then, the methods of IMA embolization, as well as the procedure itself, should be equal for all the patients (including the use of the same devices for IMA embolization, a similar level of experience of operators, etc.). 

Follow-up differed between studies, and postoperative CT angiography was performed at different time frames—most often done at 1 month, 6 months, and then annually. Still, a study with more frequent controls was also included (1, 3, 6, 12, 18, and 24 months and then annually [27]). Different methods of IMA embolization between studies could also create a potential bias. In this meta-analysis, we reported low heterogeneity, which is surprising considering that the methodology of the included studies differed widely. The main limitation of our meta-analysis is that five studies were retrospective in nature, and only one study was a randomized controlled trial [22]. The high homogeneity of our meta-analysis and concluded results suggest that IMA embolization effectively prevents type II endoleaks and secondary reinterventions. However, more prospective and randomized studies with an appropriate selection of patients based on anatomical factors are needed to prove the potential benefits of IMA embolization. 

## 5. Conclusions

The results of this study suggest that IMA embolization correlates with a lower rate of type II endoleaks and secondary reinterventions. IMA embolization performed during EVAR may be of relevance to the management of type II endoleaks; however, further prospective studies are needed to clarify this issue. 

## Figures and Tables

**Table 1:** Criteria used to perform the methodologic quality assessment.

Category	Criteria	Response		
		Yes	Partial	No
Clearly defined objective?	Clear hypothesis stated and tested. Objective easily identified in introductory section (or first paragraph of methods section). Specifies all the following: purpose, subjects/target population, and the specific association(s)/descriptive parameter(s) under investigation	x		
	Vaguely/incompletely reported (e.g., “describe the effect of” or “examine the role of”) OR substantial information must be collected from parts of the paper other than introduction/background/objective section.		x	
	Question or objective is not reported, or is incomprehensible.			x
Prospective study design?	Hypothesis designed prior to selection of participants.	x		
	Hypothesis and selection criteria designed after the occurrence of respective endpoints (e.g., occurrence of type II endoleak) Data collection conducted retrospectively after participants experienced outcome of interest (e.g., occurrence of type II endoleak)			x
Selection criteria well described?	Selection strategy designed to obtain un unbiased sample of the relevant target population. Methods for selection/recruitment/sampling detailed in the study Definition of IMA embolization adequately described (e.g., appropriate investigations of IMA through computed tomography angiography, with clinical assessment by a vascular specialist or interventional radiologist) At least three of the specified exclusion criteria described (listed next)	x		
	Selection methods (and inclusion/exclusion criteria) are not completely described OR selection methods described elsewhere. Included patients who had anatomical suitability for EVAR AND had preoperative IMA embolization AND no previous endovascular or open surgical repair Available CT angiography of patent IMA OR Available conventional angiography of patent IMA Excluded patients who had isolated iliac artery aneurysm OR ruptured aneurysms OR had fenestrated stent-grafts Excluded patients who had other methods of embolization (e.g., aneurysm sac embolization)		x	
	No information provided OR obviously inappropriate selection procedures.			x
Was an objective definition of patent IMA used?	Appropriate definition of patent IMA used, including both of the following criteria: Recognition of patent IMA by a vascular specialist/interventional radiologist IMA patency ≥3 mm	x		
	Limited definition of patent IMA described: Definition restricted to diagnosis by vascular surgeon/radiologist Definition restricted to diagnosis on imaging, but no description of anatomic characteristics of IMA		x	
	No definition of patent IMA described.			x
Assessment of outcome—Was an appropriate technical method used for IMA embolization?	Method of IMA embolization well described: Reproducibility evaluated and reported within paper AND Reproducibility determined to be moderate–high	x		
	Method of IMA embolization well described: No assessment of reproducibility reported OR Reproducibility determined to be low		x	
	Method of IMA embolization not described OR limited description provided AND no assessment of reproducibility made.			x
Sample size calculation/estimation reported in methodology?	Details of sample size calculation/estimation reported in methodology.	x		
	Required sample size reported, but no details on how this was calculated/estimated.		x	
	No sample size calculation/estimation conducted.			x
What was the sample size?	<50 OR 50–100 OR >100	N/A	N/A	N/A
	Not reported.	N/A	N/A	N/A
Did all participants undergo a CT scan prior to IMA embolization and during the follow-up?	For all patients, CT data were present both before the IMA embolization and during the follow-up.			x
Were participant characteristics adequately described?	Sufficient relevant baseline information clearly characterizing the participants is provided (or reference to previously published baseline data is provided). Includes at least five of the following: Age, sex, smoking history, hypertension, diabetes, coronary artery disease, anticoagulant use, aneurysmal diameter, number of patent lumbar arteries	x		
	Poorly defined criteria or incomplete relevant baseline/demographic information (e.g., information on likely confounders not reported). Includes fewer than five of the characteristics reported above		x	
	No baseline/demographic information provided.			x

**Table 2:** Details of excluded studies after full-text analysis; IMA—inferior mesenteric artery.

Study	Reason for Exclusion
Muthu et al. (2007) [13]	Addition of thrombin to the aneurysmal sac.
Alerci et al. (2013) [14]	Lack of control group.
Hiraoka et al. (2017) [15]	Lack of group of patients with only IMA embolization.
Parry et al. (2002) [16]	Lack of control group.
Fukuda et al. (2017) [17]	Groups of patients incompatible with our inclusion criteria; lack of separate group of patients with IMA embolization
Rokosh et al. (2021) [18]	Lack of group of patients with only IMA embolization.
Atsushi et al. (2021) [19]	Lack of group of patients with only IMA embolization.
Branzan et al. (2020) [20]	Lack of group of patients with only IMA embolization.
Nakayama et al. (2022) [21]	Lack of group of patients with only IMA embolization.
Petit et al. (2021) [5]	Lack of appropriate control group; study also involved patients who underwent fenestrated EVAR (F-EVAR).

**Table 3:** Characteristics of Included Studies. *AAA*, Abdominal Aortic Aneurysm; *CT*, Computed Tomography.

Study	Country	Study Design	Sample Size	Embolization Group	Non-Embolization Group	Inclusion Criteria for Embolization Group	Inclusion Criteria for Non-Embolization Group	Groups Matched?	Devices Used for IMA Embolization	Study Endpoints
Samura, et al., 2019 [22]	Japan	Randomized controlled trial	97	46	51	Patients with diagnosis of AAAs and anatomical suitability for EVAR, risk factors of T2EL (IMA patency with IMA ≥3 mm, LAs ≥2 mm, or an aortoiliac-type aneurysm)	The same criteria as for the embolization group; randomly assigned	No	Amplatzer Vascular Plug, metallic coils	**Primary:** occurrence of T2EL **Secondary:** maximal aneurysmal diameter change (mm), occurrence of aneurysmal sac growth, validity of defined risk factors, complications after IMA embolization, and secondary reintervention rate due to T2EL
Ward et al., 2013 [23]	United States	Retrospective cohort study	266	108	158	Patients with AAAs and a patent IMA visualized on preprocedural computed tomography (CT) angiography and subsequent conventional angiography	Patients with AAAs and a patent IMA visualized on preprocedural CT angiography but not on conventional angiography	No	coils	Incidence of T2EL, aneurysm sac volume enlargement at 24 months, and secondary interventions
Nevala et al., 2010 [24]	Finland	Retrospective cohort study	79	40	39	Patent IMA detected on computed tomographic (CT) angiography; patients at Kuopio University Hospital	Patients who underwent EVAR at Oulu University Hospital	No	Interlocking detachable coils	The presence of type II endoleak, aneurysm sac size change, and secondary procedures
Axelrod et al., 2004 [25]	United States	Retrospective cohort study	72	18	54	Patients with AAAs and a patent IMA on preoperative CT angiography + visualization of IMA on routine flush calibrated aortography	Lack of visualization of IMA on flush aortography or technically unsuccessful prior embolization	No	Platinum microcoils	The presence of T2EL, incidence of secondary procedures, and change in the diameter of the infrarenal aorta
Gould et al., 2001 [27]	United Kingdom	Retrospective cohort study	63	20	43	Patients with AAAs with assessed IMA on helical CT and calibrated angiography; the decision of final IMA embolization was made by operators	Patients with failed embolization (4), small technically unsuitable vessels (16), and with no available angiographic room time immediately before endovascular aortic repair (23)	No	Coils	The presence of T2EL, the mean sac diameter change, and secondary interventions
Vaillant et al., 2019 [26]	France	Retrospective cohort study	82	37	45	Patients eligible for EVAR (with favorable anatomical characteristics), IMA > 3 mm with no ostial occlusion or stenosis, treated after 2014	Patients treated for EVAR before 2014 with a patent IMA > 3 mm visualized on preprocedural CT scan	No	Coils, plugs	**Primary:** the rate of aneurysm sac enlargement **Secondary:** the rate of T2EL, rate of reinterventions, and overall cost of management in each group

**Table 4:** Patients’ characteristics.

	Embolization Group	Non-Embolization Group
**Samura et al., 2019 [22]**		
Age (y)	75.5	77.5
Male sex (%)	90.6%	73.6%
Aneurysm diameter (mm)	53.2	50.5
**Ward et al., 2013 [23]**		
Age (y)	73.5	75.0
Male sex (%)	88	84
Aneurysm diameter (mm)	54	56
Patent lumbar arteries	7.0	6.3
**Nevala et al., 2010 [24]**		
Age (y)	71.2	73.4
Male sex (%)	85	90
**Vaillant et al., 2019 [26]**		
Age (y)	73.78	76.73
Male sex (%)	97.3	100
Aneurysm diameter (mm)	53.19	53.07
Patent lumbar arteries	3.86	4.44

**Table 5:** Strengths and weaknesses of studies included in this systemic review. IMAE, inferior mesenteric artery embolization group.

Study	Clearly Defined Objective?	Prospective Study Design?	Selection Criteria Well Described?	Objective Definition of Patent IMA?	Well-Described Technical Aspect of Embolization?	Sample Size	Inclusion of CT Scan before IMA Embolization and during the Follow-up?	Participants’ Characteristics Described?
Samura et al., 2019 [22]	Yes	Yes	Yes	Yes	Yes	50–100	Yes	Yes
Ward et al., 2013 [23]	Yes	No	Yes	Partial	Yes	>100	Yes	Partial
Nevala et al., 2010 [24]	Yes	No	Yes	Partial	Yes	50–100	Yes	Yes
Axelrod et al., 2004 [25]	Yes	No	Yes	Partial	Yes	50–100	Yes	Partial
Gould et al., 2001 [27]	Yes	No	Partial	Partial	Yes	50–100	Yes	No
Vaillant et al., 2019 [26]	Yes	No	Yes	Yes	Yes	50–100	Yes	Yes

## Data Availability

The datasets analyzed during the current study are available from the corresponding author upon reasonable request.
